# Current and Emerging Radiotherapy Options for Uveal Melanoma

**DOI:** 10.3390/cancers16051074

**Published:** 2024-03-06

**Authors:** Oleksii Semeniuk, Esther Yu, Mark J. Rivard

**Affiliations:** Department of Radiation Oncology, Warren Alpert Medical School, Brown University and Rhode Island Hospital, Providence, RI 02903, USA

**Keywords:** uveal melanoma radiation therapy, LDR eye brachytherapy, HDR eye brachytherapy, external beam melanoma treatments

## Abstract

**Simple Summary:**

There are many possible options for treating uveal melanoma. These include brachytherapy using either low- or high-dose-rate sources temporarily implanted surgically with photon- or beta-emitting radionuclides, and also external-beam radiotherapy using high-energy linacs or with protons. In this review, we describe the various attributes and challenges for each radiotherapy option.

**Abstract:**

What treatment options are there for patients having uveal melanoma? A randomized, prospective, multi-institutional clinical trial (COMS) showed no difference in survival between brachytherapy and enucleation for medium-sized lesions. With the obvious benefit of retaining the eye, brachytherapy has flourished and many different approaches have been developed such as low-dose-rate sources using alternate low-energy photon-emitting radionuclides, different plaque designs and seed-loading techniques, high-dose-rate brachytherapy sources and applicators, and low- and high-dose-rate beta-emitting sources and applicators. There also have been developments of other radiation modalities like external-beam radiotherapy using linear accelerators with high-energy photons, particle accelerators for protons, and gamma stereotactic radiosurgery. This article examines the dosimetric properties, targeting capabilities, and outcomes of these approaches. The several modalities examined herein have differing attributes and it may be that no single approach would be considered optimal for all patients and all lesion characteristics.

## 1. Introduction

Eye cancers account for ~1% of all new cancer cases including ocular melanomas, retinoblastoma, choroidal hemangioma, and select choroidal metastases. Out of all cases, uveal melanoma (site coded as choroid, ciliary body, iris, or retina) is the most common primary intraocular cancer (~82% of all cases) arising from the uveal layer in the eye with the incidence rate of five per million annually [1]. Enucleation was the standard treatment before the 1990s until plaque brachytherapy was shown to offer comparable tumor control rates, while additionally offering patients retention of the eye and being vision sparing. Indeed, multiple studies have shown that focused radiotherapy resulted in tumor sterilization and shrinkage, thus providing an effective local tumor control, while enucleation was hypothesized to increase the risk of metastatic disease [2,3].

The purpose of this review article is to describe and compare the use of various radiotherapy options as primary treatments for uveal melanomas. Both currently available and emerging techniques in brachytherapy and external-beam radiotherapy (EBRT) are discussed. The review of ophthalmic brachytherapy includes radiation treatments with photon and electron treatments in low- and high-dose-rate (LDR and HDR) regimes. The discussion of EBRT is given for treatments using protons and high-energy photons (linac and gamma stereotactic radiosurgery).

The radiation treatment of uveal melanoma was pioneered in 1930′s by Moore and Stallard, who employed ^222^Rn and ^60^Co seeds, respectively, for brachytherapy. The next breakthrough in the field was the introduction of the low-energy photon-emitting LDR ^125^I seed, which diminished radiation exposure to others, improved the intraocular radiation distribution, and allowed for outpatient treatments. This paved the way to utilization of other low-energy seeds arranged inside a high-Z backing, including ^103^Pd and ^131^Cs [4,5]. Beta-emitting ^106^Ru/^106^Rh and ^90^Sr/^90^Y radionuclides also became available and gained popularity, especially in Europe for both HDR and LDR treatments [6,7].

## 2. Molecular Pathogenesis of Uveal Melanoma

The molecular pathogenesis of uveal melanoma is increasingly being elucidated, and tumor genetic factors have been correlated with patient prognosis. Driver mutations in GNAQ/GNA11 appear to be driver mutations in the vast majority of uveal melanoma cases, though do not necessarily correlate with survival outcomes. These genes encode proteins involved in the RAS-RAF-MEK-ERK- (MAPK) pathway which is critical to cell growth and proliferation [8]. Dysregulation of other cellular pathways autophagy has also been implicated in uveal melanoma tumorigenesis [9]. Cytogenetic rearrangements in uveal melanoma most commonly affect chromosomes 1, 3, 6, and 8 and can predict the risk of poor outcomes. Monosomy 3 and 8q gain are known to predict an increased risk of development of distant metastasis. A concurrent loss of 1q with monosomy 3 predicts poor disease-free survival whereas a gain of 6p appears to portend a favorable prognosis. Alterations in tumor-suppressor gene function, in particular BAP1 located on chromosome 3, have been shown to correlate with increased risk of tumor metastasis. Mutations in EIF1AX occur in 8% to 19% of uveal melanomas and affect protein translation and correlate with improved prognosis and low metastatic potential. SF3B1 mutations which affect spliceosomes are more often found in younger patients and seem to carry an intermediate risk of metastasis which can develop several years after diagnosis. The various molecular mechanisms of tumorigenesis and the development of metastatic potential are attractive targets for a range of systemic therapies and remain under active investigation [8,9].

## 3. Clinical Workup

Workup and evaluation for uveal melanoma include a detailed history and physical examination. The American Joint Committee on Cancer tumor-staging guidelines for choroidal and ciliary body melanomas depend on tumor thickness, largest basal diameter, presence of ciliary body involvement, degree of extraocular extension, and the presence of regional and/or distant metastases. When organ preservation is feasible, options for definitive radiation treatment modality may depend on tumor size and thickness and possible involvement of the optic nerve. Implementation of definitive radiotherapy requires multidisciplinary input and coordination between an experienced team of ophthalmologists, radiation oncologists, medical physicists, anesthesiologists, and radiation safety staff.

In general, the surgical risk and the patient’s candidacy for general anesthesia and their proximity to high-volume plaque brachytherapy and/or proton centers can influence treatment modality selection in addition to tumor characteristics. Lesions less than 19 mm in diameter and up to 10 mm thick can be considered for definitive treatment via plaque brachytherapy or particle beam radiation. The choice between brachytherapy vs particle beam radiation can also depend on specific tumor characteristics (e.g., tumor size, ocular location, proximity to the optic nerve) and institutional experience in treating many patients with one or both modalities. Lesions that are >10 mm in diameter, >10 mm thick, or lesions that are close to or involving the optic nerve are typically considered for particle beam radiation, stereotactic radiosurgery, or enucleation especially for tumors with extensive extraocular extension [10]. A summary comparison of radiation treatment techniques for uveal melanoma with associated tumor control and complications is given in Table 1.

## 4. Brachytherapy

Brachytherapy is administered for uveal melanoma by affixing LDR radioactive sources/seeds to a brachytherapy plaque that is surgically secured temporarily to the globe. Plaque immobilization to the globe near the tumor eliminates the risk of a geographic miss due to ocular mobility as long as the plaque is placed properly at the time of surgery. The rapid radiation-dose fall-off beyond the target allows for minimal adverse effects to the eye adjacent to the target.

Overall, outcomes with plaque brachytherapy are favorable generally irrespective of the radionuclide [11,12]. Buonanno et al. report 5-year local tumor control of 95% in a compiled cohort of 21,263 patients treated with plaque brachytherapy, including a variety of plaque sources including ^125^I, ^106^Ru, ^60^Co, ^192^Ir, and ^131^Cs. Incidences of distant metastasis and enucleation were reported to be 8% and 7%, respectively. Adverse effects of plaque brachytherapy include acute risks of anesthesia and surgical risks of infection, bleeding, and discomfort and/or injury to intraocular muscles or periorbital soft tissues. Late effects also occur at variable frequencies. Cataracts are common with an incidence of 20% [16]. Radiation retinopathy may develop first as a non-proliferative occlusive vasculopathy which can subsequently progress to vision loss through variable ischemic necrosis. Risk of this complication can depend on comorbidities including diabetes, hypertension, radiation dose, and proximity of the tumor to the foveola. Radiation retinopathy is commonly treated with photocoagulation, vitrectomy, and observation. Radiation retinopathy specific to the macula is known as radiation maculopathy and has been reported in 25% of patients after radiation [17]. Radiation maculopathy is typically treated with anti-VEGF intravitreal therapy and intravitreal steroids implants. Uveal melanomas can disrupt the attachment of the retina and sclera and can commonly cause vitreous hemorrhage, but radiation can affect the local vasculature leading to ischemia and neovascularization and thereby increase the risk of vitreous hemorrhage and retinal detachment. The 5-year incidences for vitreous hemorrhage and retinal detachment have been reported as 18% and 2%, respectively, in patients undergoing ^125^I plaque brachytherapy [18,19]. Secondary glaucoma is another late complication that may occur within a few years following brachytherapy and has been reported in 23% of patients. Mechanisms of increase in intraocular pressure may involve the tumor location obstructing the chamber angle and/or neovascular glaucoma. Additional risk factors include tumor thickness, retinal detachment, and primary underlying elevated intraocular pressure prior to radiation [20]. Scleral necrosis is a rare complication in 1% to 5% of cases where the risk is associated with an increasing radiation dose and tumor thickness [16,21].

### 4.1. LDR Brachytherapy with Low-Energy Photons

**Figure 1:**
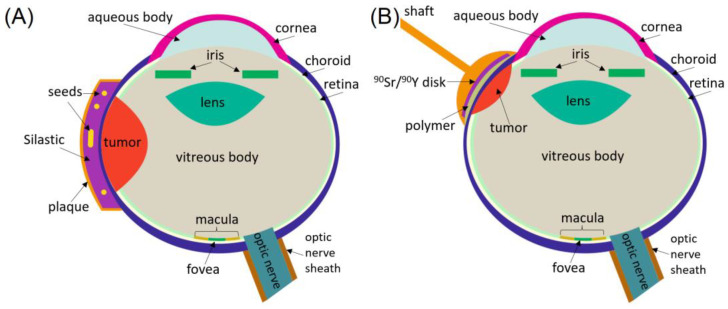
(**A**) Cross-sectional view of the eye with the LDR COMS plaque. (**B**) HDR beta-particle treatments with a hand-held applicator.

The majority of LDR photon brachytherapy is performed with low-energy photon-emitting seeds assembled in a high-Z episcleral plaque as shown in Figure 1A. The Collaborative Ocular Melanoma Study (COMS) plaques are most widely used and are made of gold-alloy backing. They are available in a variety of sizes ranging from 10 mm to 22 mm in diameter (2 mm increments) and contain from 5 to 24 seeds. The seeds are placed within the grooves of the bio-compatible Silastic insert (MDX4-4210 Dow Corning Corp., Midland, MI, USA) [4,22,23]. More diagrams of these applicators and their geometrical dimensions can be found in the AAPM TG-129 and TG-221 Reports [4,24]. For treatments of tumors abutting the optic nerve, a plaque is used having a notch with those seeds removed. This allows the densely-loaded plaque region to be more central to the tumor, thus providing improved coverage, while the gold-alloy wall of the plaque reduces irradiation of the optic nerve [25].

While Silastic-based plaques currently dominate eye plaque brachytherapy, it is a low-Z material that results in nearly cylindrically-symmetric dose distributions, shielded only by the gold-alloy backing. Such dose distributions result in the sclera receiving higher radiation dose than the target. An alternative Silastic-free plaque design is gaining popularity, where the seeds are held in place within grooves of the gold-alloy backing. Being high-Z material, these grooves act as collimators to provide miniature radiation beams from each seed. The beams from adjacent sources combine at depths beyond a millimeter into the tissue, uniquely offering scleral dose sparing with a potential reduction in radiation retinopathy rates for treatments at the posterior pole [2,26].

Historically, the prescription dose evolved from 100 Gy to 85 Gy to the tumor apex, with physical doses to tissue and accounting for realistic plaque materials being an additional 25% lower [4,27,28]. Treatments are delivered over 3 to 7 days [29,30]. Since eye cancers are relatively rare (accounting for <1% of all new cancers), a typical cancer center may have only a few patients per year. Generally, a new batch of seeds of a single source-strength is ordered for each case since radioactive decay over the interval between patients would prohibit their reuse [31]. However, busier cancer centers with higher referral rates of uveal melanoma patients have adopted a non-uniform loading of the plaques to make the treatment cost efficient and more dosimetrically optimal [32,33].

### 4.2. LDR Brachytherapy with Beta-Particles

Modern LDR beta particle-emitting plaques typically utilize a reusable ^106^Ru/^106^Rh source in a silver shell (Eckert & Ziegler BEBIG, GmbH, Berlin, Germany). Unlike COMS plaques using Silastic and gold alloy, ^106^Ru/^106^Rh sources come in thin layers that are electrodeposited onto the concave surface of a silver backing. A 0.1 mm thick silver exit window prevents direct patient contact of the radionuclide [34]. Similar to COMS plaques, notched plaques for treatment of juxtapapillary tumors are used to reducing the dose to the optic nerve or iris. A sketch of these applicators and their geometrical dimensions can be found in ICRU Report 72 [4,35]. ^106^Ru/^106^Rh applicators are designed to deliver a radiation dose of ~100 Gy to the tumor apex over a period of 4 to 7 days [36].

### 4.3. HDR Brachytherapy with Photons

Overall, uveal melanoma brachytherapy is dominated by LDR brachytherapy. Nevertheless, its HDR counterpart is under active research and development for both photon-emitting and beta-particle options. HDR brachytherapy is based on an applicator and a remote afterloader with the radioactive source. ^192^Ir is the most common radionuclide due to its combination of high penetration depth, relatively long half-life, and high specific activity. The Freiburg flap, Valencia, and Leipzig applicators are all used to treat superficial skin lesions while generic and modified (with interstitial needles) cylindrical applicators are used for treatment of cervical tumors [37]. Recently, Dupere and colleagues have proposed expanding the utilization of HDR brachytherapy by treating choroidal melanomas with an HDR ^169^Yb source in conjunction with a gold shielded applicator. In their proposal, the applicator has a ring-like channel for the guiding of the source. The channel is collimated to provide a divergent radiation beam. Applicator diameters (12 mm to 18 mm with 2 mm increments) and radiation divergence angles are selected to provide optimal tumor coverage while allowing for dose sparing of radiosensitive adjacent structures. The ^169^Yb source is enclosed in a titanium capsule that is welded to a stainless-steel cable driven by the afterloader, which allows for remote source deployment to the desired dwell positions and radioprotection of hospital staff. Further descriptions of the modality and applicator geometry are found elsewhere [38,39]. Biologically equivalent to 85 Gy from LDR, an HDR prescription of 30 Gy is delivered in single fraction over 10 min as opposed to ~5 days for LDR brachytherapy [39].

### 4.4. HDR Brachytherapy with Beta-Particles

HDR ocular brachytherapy with beta-particles was conventionally performed with a ^90^Sr applicator with ^90^Sr and ^90^Y in secular equilibrium. The original device was hand-held and composed of a stainless-steel shaft with a 16 mm diameter concave surface applicator (SIAQ 7321, Amersham Corporation, Amersham, UK). ^90^Sr was coated on a concave side of the applicator so the radioactive zone had a diameter of 12 mm as shown in Figure 1B. In the modified version, the shaft was replaced by a stainless-steel ring applicator into which the radioactive plaque was placed. Further details on the construction of the ^90^Sr/^90^Y devices can be found elsewhere [7,38,40]. With the original shaft-based device, a dose of 75 Gy was delivered over 15 to 20 fractions of ~10 min each. With the ring applicator, the treatment was delivered in a single 2 h fraction. During this time, the patient remained in the operating suite [38]. The ^90^Sr half-life is 28.9 years, which allows utilization of the same device over decades.

Modern HDR beta-particle treatments are based on a single radionuclide ^90^Y source in a hand-held applicator (Liberty Vision, Portsmouth, NH, USA). In contrast to the original ^90^Sr-based devices, the new ^90^Y sources are made in 1-mm flat discs with 6, 8, or 10 mm diameters. Due to the 64 h half-life of ^90^Y, each radioactive source is intended for a single use with the acrylic handle designed for source exchange. Treatments are about 10 min, during which a ~30 Gy dose is delivered to the tumor apex [41].

## 5. External Beam Radiation Therapy

### 5.1. Gamma Stereotactic Radiosurgery

Gamma stereotactic radiosurgery via Gamma Knife (GK-SRS) using ^60^Co (5.27 year half-life) is also an effective treatment modality for larger ocular melanomas [14,42,43,44,45,46,47]. GK-SRS planning requires accurate tumor delineation on CT or MRI, thereby making treatment of small tumors more difficult. GK-SRS is especially useful for treatment of large lesions that are not amenable to plaque therapy [42,43]. Adequate immobilization of the eye for the duration of treatment is critical. While tumor control with GK-SRS is generally favorable, vascular complications with secondary glaucoma are common and reported in up to 47% of patients [48].

GK-SRS using a stereotactic headframe was originally designed to treat intracranial lesions, and over the years has proven its efficacy to address various conditions such as meningiomas, schwannomas, acoustic neuromas, and arteriovenous malformations [49]. Some of the most important features of GK-SRS, such as steep dose fall-off and the possibility to deliver high radiation doses to a confined area, make it also attractive for ocular radiosurgery. In comparison with brachytherapy options, GK-SRS has an advantage of being able to treat tumors of heights >10 mm [49]. Further, ocular melanoma cell lines can be relatively radioresistant (equally or less susceptible to radiation than adjacent normal structures) in vivo, especially at lower doses, and they will nonetheless respond to single high doses delivered by stereotactic radiosurgery or high-dose brachytherapy [50,51]. GK-SRS treatments are relatively long and take 2 to 4 h to deliver a dose of 30 to 50 Gy, prescribed to the 50% isodose surface encompassing the planning target volume (PTV) [50,52,53].

### 5.2. Linac EBRT

Outcomes from treatment of ocular melanomas using linear accelerators are also promising. A series of 24 patients treated with fractionated stereotactic radiation used a prescription dose of 60 Gy. With a median follow-up of 5.2 years, 82% of the patients remained alive and without evidence of local progression, but with an enucleation rate of 23% [54]. No grade 4 acute toxicities were seen, though 25% of patients ultimately developed Grade 4 ipsilateral retinopathy resulting in blindness. Another linear accelerator-based radiosurgery method includes CyberKnife, which is a robotically-controlled linac. CyberKnife results also demonstrate good results. The largest retrospective series included 594 patients treated with a single fraction, and observed 5-year local control of 84% with 81% eye retention when treated to 22 Gy [15]. Retinal detachments and secondary glaucomas were observed in 36% and 17.5% of patients.

The major disadvantage of Gamma Knife is the limited number of treatment fractions (usually a single fraction) due to the invasive nature of patient immobilization and retrobulbar block, which is further discussed in Section 6.2. Thus, the radiobiological advantage of fractionated treatment is not utilized. A number of studies have shown that stereotactic radiotherapy (SRT) performed with linacs and relocatable frames/modified thermoplastic masks can be an alternative treatment modality of ocular melanomas. Fractionated linac SRT reduces the effective dose to surrounding tissues, yet delivers an equivalent effective dose to the tumor. In addition, the relocatable frame or thermoplastic mask is less invasive and more convenient [55,56,57,58].

Treatments are performed with a 6 MV photon beam and tertiary circular collimators (10 to 45 mm in diameter) as required to achieve steep dose gradients [58,59]. It should be noted that dose gradients for GK-SRS, proton beam therapy, and brachytherapy are generally steeper than those achievable in linac-based SRT with penumbras of about 2 mm versus 6 mm [58,60,61]. A dose of 50 Gy to 70 Gy prescribed to the 80% isodose is applied to the tumor in five fractions [55,56,57,58]. Depending on target size and shape, one or two isocenters are used for treatment delivery with 4 to 7 arcs per isocenter [58,59].

### 5.3. Protons

Oncologic outcomes with proton beam radiation are excellent. In a series of 336 patients with mean basal tumor diameters and heights of 18.2 mm and 8.2 mm, treatment of 70 Gy in 5 fractions with protons resulted in tumor control of 87.5% at 10 years with 70.4% of patients retaining the eye and baseline visual acuity of 20/200 or better in 72.6% of patients [13]. These were similar to additional studies of patients receiving proton beam radiation [62,63,64]. Complications after proton beam radiation are variable and include glaucoma (7% to 30%), cataracts (20% to 62%), vitreous bleeding (9% to 14%), retinopathy (23% to 67%), and optic neuropathy (33%) with risks depending on location of size/tumor and treatment dosimetry. These toxicities can be treatable with a combination of surgical and/or medical interventions such as aqueous shunt placements and/or ranibizumab for glaucoma, bevacizumab and laser photocoagulation for retinal detachment and anterior segment neovascularization, and intravitreal corticosteroids for maculopathy [65]. There are no large-volume, randomized data showing clear superiority of protons over plaque brachytherapy, and issues of selection bias limit comparison of outcomes across differing patient series.

Large tumors, particularly those located close to the optic nerve, are difficult to treat with plaque or EBRT due their proximity to those structures most relevant for preservation of visual acuity (i.e., optic nerve, macula, ciliary body). In general, tumors located within 2 mm of the optic disk or fovea centralis should not be treated with radioactive plaques due to the high doses at areas in close proximity to the surface of the plaque applicator and hence an inevitable risk of radiation damage to these structures, leading to development of radiation maculopathy or optic neuropathy as well as increased risk of local failure [66,67,68,69]. Collimated protons deliver a maximum dose at the end of their tracks, referred to as the Bragg peak effect. This effect allows a reduction in the target exit dose to nearly zero within a couple of millimeters, which is not achievable with alternative plaque or EBRT methods [70,71,72,73]. The Bragg peak depth can be broadened by varying or modulating the beam energy to allow dose distribution conformity to any tumor shape and depth. As a result, protons allow for highly localized and uniform dose distributions (needed for target coverage) with a sharp dose fall-off outside the treated area, which in its term provides a superior healthy tissue-sparing effect [74,75,76,77,78,79,80].

Proton beams of about 65 MeV are typically produced by cyclotron accelerators. Compared to ^60^Co irradiation, protons have a relative biologic effectiveness (RBE) of ~1.1. Therefore, proton doses are typically given as Cobalt Gray Equivalents (CGE) and in general they are lower than the current standard of 85 Gy to water-in-water for plaque brachytherapy. Thus, patients treated with protons received 60 to 70 CGE to the tumor apex delivered in five fractionations over a 7- to 14-day period [75,81,82,83,84].

## 6. Target Imaging, Patient Immobilization and Treatment Delivery Verification

Patient imaging and disease localization are the fundamental steps of any radiotherapy treatment. Conventional radiation therapy typically relies heavily on imaging modalities like computed tomography (CT), positron emission tomography (PET) (usually in combination with CT), single photon emission tomography (SPECT), and magnetic resonance imaging (MRI). While useful for tumor staging and the detection of distant metastases, the major drawback of the aforementioned modalities for eye imaging is the relatively large voxel size in comparison to ocular structures. For example, the fovea is ~0.05 mm in diameter, much smaller than CT or MRI voxel dimensions, and cannot be distinguished from adjacent structures. Therefore, alternative modalities capable of submillimeter resolution such as ultrasound (US), fluorescein angiography ophthalmoscopy, scleral transillumination, and fundus photography are used to facilitate treatment planning of uveal melanomas for measurements of tumor profile and dimensions [7,38,39,85]. Posterior melanoma diagnosis is particularly challenging, since it can be confused with a number of alternative conditions that include retinal lesions, retinal pigment epithelium, circumscribed choroidal hemangioma, and age-related macular degeneration. In order to eliminate the diagnosis ambiguity, a fine-needle aspiration biopsy of a uveal tumor can be performed by the ocular oncologist [86].

### 6.1. Brachytherapy

During brachytherapy, the tumor location is confirmed with US/ophthalmoscopy/ transillumination and identified with a surgical marker. The desired plaque size is ~2 mm larger than the tumor size to account for positioning uncertainties and microscopic disease extension beyond the visible tumor edge [2,4,5,7,24,38,39,85].

COMS and BEBIG plaques are applied preloaded with sources. Therefore, all attempts are made to position these plaques on the sclera precisely over the tumor, and then suture it to the sclera in minimal time in order to reduce medical staff exposure. The modified ^90^Sr applicators are more forgiving in this aspect since a hollow ring is first sutured to the sclera, and the ^90^Sr plaque is then placed into the applicator ring, which allows reduced manipulation time of the radioactive source of only several seconds [38]. The ^169^Yb ring applicator is first sutured to the eye and then connected to the remote afterloader, thus resulting in no plaque installation dose [39]. After the plaque is affixed, any eye movements are not thought to affect plaque-dose distribution [2]. One of the advantages of episcleral plaque treatments over shaft-based (^90^Sr/^90^Y and ^90^Y) counterparts is their larger treatment areas. Indeed, the maximum diameter of a COMS plaque is 24 mm, and BEBIG plaques have diameters up to 25 mm, which is more than a factor of two larger than the size of the ^90^Y applicator having a maximum diameter of 10 mm. Thus, the treatment of tumors larger than the plaque diameter requires a shift, i.e., extra treatments to treat the uncovered areas [7,38]. In general, when compared to photon-emitting applicators [87], treatment outcomes with beta-emitting applicators are more sensitive to plaque placement and require deep experience for adequate target coverage due to a sharp beam penumbra that is more sensitive to a geographic miss [4,24].

Naturally, plaque-based techniques require a second surgery to remove/explant the plaque. This is not the case though with the shaft-based HDR brachytherapy as with ^90^Y. Indeed, with the patient in a supine position, the applicator is hand held with the shaft over the treatment on the marked ocular surface. The shaft is made of semi-transparent acrylic to allow real-time visual monitoring of the target position. The surgery is needed only to temporarily reposition any obstructing muscles. The check for a clear pathway to the desired plaque position is verified with a non-radioactive applicator and photography of directional lights embedded in the applicator holder [41]. The latter provides a great advantage over plaque-based techniques since metal plaques are opaque and position verification is non-trivial.

The aforementioned imaging methods are used mostly for tumor characterization and treatment planning. The presence of a high-Z plaque backing can create significant imaging artefacts in CT due to photon attenuation in the plaque. In addition, CT does not produce high-quality soft tissue contrast, which makes target delineation challenging [24,88,89]. US has become a method of choice for implantation guidance and post-verification imaging. One of the drawbacks of US for uveal melanoma imaging is that it cannot generate a dataset, i.e., a collection of parallel slice images that can be used for post-implantation dose calculations [88]. On the other hand, MRI imaging has excellent soft tissue contrast and can readily provide such volumetric imaging. In addition, due to the low magnetic susceptibility of the gold alloy, the images are free from streaking artifacts that are inherent to CT. One concern with MRI of the plaque is the diamagnetic properties of gold, a major component in the COMS alloy. Gold does not enhance under MRI imaging and appears as a void, causing a susceptibility artefact that obscures the patient’s anatomy near the plaque [24]. Recently, Zoberi and colleagues have developed and evaluated an MRI technique to address this issue. The technique uses prior MRI of a gel phantom with the eye plaque and a combination of T2-weighted, T1-weighted, and proton-density weighted MRI sequences to deduce the optimal scan parameters for adequate visualization of the anatomy pre- and post-implantation of the plaque with any detected plaque shifts and tilts of ~1 mm [89].

### 6.2. EBRT

While patient imaging for treatment planning with EBRT is similar to that for brachytherapy, the requirements of treatment delivery are more stringent since the radiation source is not affixed to the tumor and eye movement could result in a geographic miss. Therefore, patient and eye immobilization are of paramount importance for successful treatment delivery. This is especially necessary with GK-SRS since treatments last hours to deliver a therapeutic dose in a single fraction. Thus, patient immobilization starts with attaching a stereotactic headframe with four pins in such a way that the target is approximately centered with respect to the stereotactic system. The eye is immobilized by a retrobulbar anesthesia block to obtain complete akinesia. Ophthalmic lubricant ointments are applied to the involved eye periodically to prevent corneal dryness. Once the patient is immobilized, high-resolution (≤2 mm slice thickness) MRI with gadolinium contrast is acquired for target delineation and treatment planning, while CT (having electron density data) is used for dose calculations [49,50,51,52,53]. PTV margins of 2 mm are applied around the tumor in uveal melanoma treatment with GK-SRS, linac, and protons [49,50,55,56,58,90].

**Figure 2:**
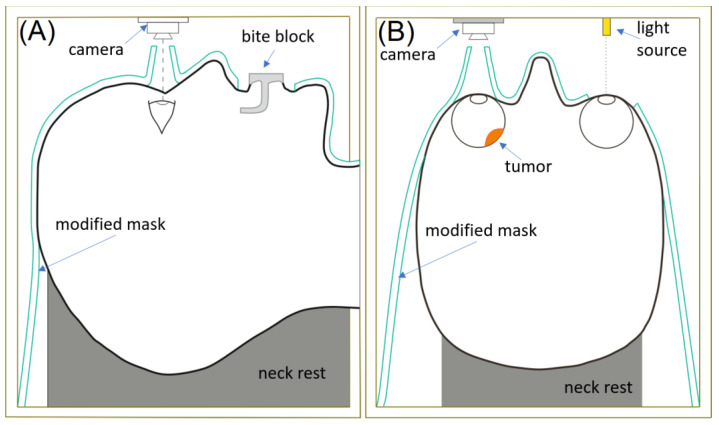
Patient immobilization and eye tracking during EBRT. (**A**) Sagittal view. (**B**) Axial view.

For linac-based irradiation of ocular melanomas, a somewhat different procedure was established to account for fractionated treatment. One of the major modifications of patient immobilization lies in the utilization of relocatable frames or a thermoplastic mask, which are more comfortable for the patient (Figure 2). The procedures are shorter (30 min with linac vs. 2 to 4 h with GK-SRS) which replaces eye immobilization with focusing the patient’s gaze on an optic beacon located 0.2 m away. At the same time, a miniature camera is used to monitor the involved eye. If eye motion is detected above an established threshold, the treatment beam is interrupted until the patient’s gaze returns to the desired position [56,57,58]. Cutouts made into the thermoplastic mask accommodate the optical monitoring. In order to use the system during MRI, a camera was enclosed in a grounded copper box [57]. Further the mask was modified to include the nasal bridge and bite blocks for improved head immobilization [57]. As such, linac treatments with a thermoplastic mask and optical monitoring are the only non-invasive treatment modality for uveal melanoma [57,58].

Protons also use optical eye position fixation and monitoring. However, tantalum marker clips or carbon fiducials are also used for treatment planning and radiosurgery guidance [91]. These markers are sutured to the sclera at the tumor periphery, delineated by transillumination and indirect ophthalmoscopy [7,70,75,81,82,83]. The patient’s head is immobilized with a bite-block and a modified facemask to access the involved eye [75,81,83]. Before treatment, the patient and target eye are aligned with the beam using fluoroscopic or orthogonal imaging of the markers [75,83]. Imaging is also performed if the patient moves or deflects the eye from the optic beacon.

## 7. Radiation Safety

The ^125^I, ^103^Pd, and ^131^Cs sources of COMS LDR plaques emit low energy photons of 28.4, 20.7, and 31 keV, respectively. The sources pose radiation concerns mostly during implantation, leading to a hand dose of approximately 5 mSv to the surgeon per surgery [39,92,93]. Post-implantation, however, the plaque radiation is easily shielded by 0.5 mm of gold-alloy backing. This allows the treatment to be delivered in the outpatient fashion. There are no special radiation safety precautions for the patient or the public. A high-Z patch can be worn while the plaque is in place to limit the public exposure even further, but this is not mandatory [4,24]. It is strictly mandatory for the patient to come back to the hospital on the scheduled day for the plaque removal. Delay of the plaque removal could result in patient overdose and cause exacerbated side effects. Therefore, weather and/or transportation-related factors, limiting patient access to the hospital, should be considered upon selection of the optimal treatment modality.

Care should be taken with ^106^Ru beta-emitting LDR plaques to make sure that the 0.1 mm Ag layer separating the radioactive coating and the patient is still in place [34]. For this, careful wipe tests need to be performed regularly.

^169^Yb emits ionizing photons in the range of 50 to 308 keV, with a median energy of 58 keV and an average energy of 93 keV. An applicator containing a backing of only 1.2 mm thick gold is calculated to reduce the absorbed dose by 90% while a 2 mm backing will reduce the dose by 96%. Because of the relatively low energies of ^169^Yb photons and the effectiveness of a lead shielding (10 mm is required), the operator can be adjacent to the patient during treatment. Furthermore, since HDR ^169^Yb-based devices use an afterloader, the surgeon sutures and removes an empty applicator thereby resulting in no hand dose [39].

The design of the ^90^Sr/^90^Y and ^90^Y shaft-based devices includes protective acrylic shields to reduce the physician’s exposure [40]. When the physician’s fingers are positioned close to the involved eye, it is suggested that additional radiation protection could be obtained by filling a surgical glove with water.

With the EBRT solutions, all patient preparatory work (eye immobilization, patient positioning) is performed with the beam off. Therefore, from a radiation protection standpoint, EBRT modalities are the safest options for hospital staff.

## 8. Future Directions

Similarly to the advancements and variety of the treatment delivery options, the dose calculation algorithms are being actively investigated. Traditionally, dose calculation in brachytherapy is performed following the AAPM TG-43 formalism [94]. While the TG-43 method is approved by the U.S. Food and Drug Administration, Health Canada and the European CE Mark, and incorporated in a number of treatment planning systems (TPSs) such as Pinnacle^3^ (Philips Medical Systems, Cleveland, OH, USA) and BrachyVision (Varian Medical Systems, Palo Alto, CA, USA), it has certain limitations. Particularly, it assumes homogeneous water medium composition of both the plaque and the patient and ignores the interseed effects [94]. This results in dose overestimation in some cases exceeding a factor of ten [23,27,95]. Currently, there are no governmentally approved TPSs that account for plaque attenuation, interseed effects and patient heterogeneity. This topic is being actively investigated. Non-FDA-approved TPSs include analytical- and Monte Carlo (MC)-based solutions. The AAPM TG-221 report addresses the dosimetric effect of the plaque where correction factors are available for a limited number of seed models (some are obsolete) and medium heterogeneity remains ignored. Plaque Simulator^TM^ (PS) (Eye Physics, LLC, Los Alamitos, CA, USA) uses a plaque and interseed heterogeneity correction factor for a wide variety of COMS plaques; however, water remains the calculation medium. MC-based solutions intrinsically account for all corrections required. However, this comes at a high computation cost, requiring unique user skill in simulation modeling and validation, dedicated equipment (computer clusters), and current calculation times of about one day [23,96,97]. MC simulations fostered the development of the hybrid solutions (for instance, open-sourced EyeDose), which leverages pre-computed MC dose distributions [98], to calculate the dose in the target and various eye structures. Unfortunately, currently this has been realized only for standard water-equivalent eye models with generic COMS plaques. The future advancements in the field of uveal melanoma treatments are set to account for a patient-specific heterogeneous anatomy and variety of generic and notched eye plaques. In addition, the dose verification is envisioned to be performed on post-implantation imaging, similarly to how it is implemented for post-operative LDR prostate treatments [99]. In addition to the improved dose reporting, such advancements will prompt rather larger changes in the whole field of brachytherapy, fostering transition from prescription to medium-in-medium as opposed to the status quo water-in-water. This has already been implemented in the online adaptation treatment platform Ethos (Varian, Palo Alto, CA, USA) for EBRT [100]. While the retrospective analysis of the patient outcomes with the historical data might be challenging, this might pave the way to the development of new treatment regiments. For instance, the optic cord dose is one of the major concerns in dose escalation in eye radiation therapy, as it causes irreversible vision loss [101]. This makes the coverage of deep-seated tumors abutting the optic nerve particularly challenging, especially with brachytherapy. Since currently used TG-43-based TPS systems tend to overestimate the dose [23,27,95], the precise dosimetry might allow dose escalation for achieving optimal patient outcomes.

## 9. Conclusions

There are many effective forms of radiotherapy for uveal melanoma. The optimal form of treatment has not yet been discovered. The several modalities examined herein have differing attributes and it may be that no single approach would be considered optimal for all patients and lesion characteristics.

## Figures and Tables

**Table 1:** Comparison of radiation treatment techniques for different tumor conditions.

Radiation Technique	Tumor/Treatment Considerations	Radiation Modality	Dose	Local Control (LC)	Complications
Radioactive seed plaque brachytherapy	Diameter < 19 mm, thickness < 10 mm, >2 mm away from optic nerve, surgery/anesthesia risk	Radioactive seeds (^125^I, ^106^Ru, ^60^Co, ^192^Ir, and ^131^Cs)	~85 Gy in 3–7 days	5 yr LC 90–95% [11,12]	Cataracts, radiation retinopathy, vitreous hemorrhage, glaucoma, scleral necrosis
Proton beam radiation	Proximity to optic nerve, eye immobilization	Protons	70 Gy in 5 fractions	10 yr LC 80–90% [13]	Cataracts, glaucoma, vitreous hemorrhage, retinopathy, optic neuropathy,
GK-SRS	>2 mm from optic nerve, eye immobilization	^60^Co	20–50 Gy in 1 fraction	1–5 yr LC 85–95% [14]	Glaucoma, retinopathy, vitreous hemorrhage
Linear accelerator-based radiosurgery	Proximity to optic nerve, eye immobilization	Photons	~22 Gy in 1 fraction, 60 Gy in 3–5 fractions	5 year LC 80–85% [15]	Cataracts, radiation retinopathy, vitreous hemorrhage, glaucoma

## Data Availability

The authors freely make available any of the data in this article.
